# Response to commentary ‘long COVID-19/post-COVID condition in children: do we all speak the same language?

**DOI:** 10.1186/s13052-022-01394-4

**Published:** 2022-12-19

**Authors:** Gianfranco Trapani, Giuseppe Verlato, Enrico Bertino, Giulia Maiocco, Roberta Vesentini, Alessia Spadavecchia, Angelica Dessì, Vassilios Fanos

**Affiliations:** 1 Alfred Nobel International Association Studies Center, Sanremo, Italy; 2 Unit of Epidemiology and Medical Statistics, Department of Diagnostics and Public Health, University of Verona, Verona, Italy; 3 Department of Public Health and Pediatric Sciences of the University, City of Health and Science of Turin, Turin, Italy; 4 Department of Surgical Sciences, University of Cagliari and Neonatal Intensive Care Unit, AOU Cagliari, Cagliari, Italy

**Keywords:** Long COVID-19, post-COVID condition, Pediatric age

## Abstract

Here we present the Authors’ answer to the Letter written by Dr. Garazzino and Colleagues with reference to our article “Long COVID-19 in children: an Italian cohort study”.

## Main text

Dear Editor,

we read with interest the letter by Dr. Garazzino and colleagues. They present a large hospital series with a cumulative incidence of post-COVID condition (PCC) of 8.4% (35/417), in contrast with the incidence recorded in our cohort of outpatients visited in primary care, which was of 24.3% (153/629) [1].

Of course, it is useful to try to analyze the possible reasons that could explain these differences.

The first point to consider is the difference existing between the two studies regarding design and follow-up. The Turin study has a prospective design, whereas our study is cross-sectional. Moreover, the first refers to a monocentric, hospital-based series, whereas the second is a multicentric study which considers mainly primary care patients. Multicentricity could be surely an added risk of inhomogeneity in evaluation of PCC symptoms, especially when self-reported and regarding qualitative and subjective conditions like fatigue or psychological disorders. We tried to contain this risk by directly making the Primary Care Pediatrician detect symptoms during the interview with the parents or during the clinical visit.

A very important role is played by the differences in the PCC definition itself.

Dr. Garazzino and colleagues deemed that only symptoms occurring or persisting within 12 weeks from SARS-CoV-2 infection were consistent with sequelae of COVID-19. In our study instead, long COVID-19 syndrome was diagnosed when at least one of the symptoms increased in frequency during the 8–36 weeks after recovery from SARS-CoV-2 infection, with respect to the previous year.

Another reason could be that the two study cohorts have been recruited in two different time periods. The series from Regina Margherita Children’s Hospital was collected in March 2020 – January 2022, while our survey was performed on patients who acquired SARS-CoV-2 infection in October 2020 – June 2021. Hence, most children in our series had COVID-19 before the vaccination era, while severity of COVID-19 greatly decreased after the start of vaccination and the surge of *omicron* variant in the second semester of 2021.

In addiction with the primary care series, we also presented data of a small hospital series regarding 60 children admitted at the Vittore Buzzi Children’s Hospital in Milan, Italy. PCC cumulative incidence in this cohort is higher: the previously mentioned reasons could have a role in the explication of the difference.

In our opinion, research in the hospital setting and in primary care are both essential, even if they yield different results as in the present situation. Hospital series are better characterized through medical specialist visits, lab exams, imaging.

Anyway, we completely agree with the conclusions, reported by Dr. Garazzino and coll.: “PCC in the pediatric population is still an ill-defined entity that urgently needs agreed and standardized definitions, as well as outcome measures. Only when these items are conclusively established, the real prevalence of the disease will be clarified”.

## Data Availability

The datasets used and/or analyzed during the current study are available from the corresponding author on reasonable request.

## Abbreviations

SARS-CoV-2: Severe Acute Respiratory Syndrome CoronaVirus 2
COVID-19: CoronaVirus Disease 2019
